# Translation into Brazilian Portuguese and transcultural adaptation of the Apraxia of Speech Rating Scale 3.5

**DOI:** 10.1590/2317-1782/20232022012en

**Published:** 2023-06-30

**Authors:** Dhébora Heloísa Nascimento dos Santos, Ivonaldo Leidson Barbosa Lima, Leonardo Wanderley Lopes

**Affiliations:** 1 Programa Associado de Pós-graduação em Fonoaudiologia, Universidade Federal da Paraíba - UFPB - João Pessoa (PB), Brasil.; 2 Departamento de Fonoaudiologia, Universidade Federal do Rio Grande do Norte - UFRN - Natal (RN), Brasil.; 3 Departamento de Fonoaudiologia, Universidade Federal da Paraíba - UFPB - João Pessoa (PB), Brasil.

**Keywords:** Apraxias, Speech Disorders, Differential Diagnosis, Validation Study, Psychometry, Speech, Language and Hearing Sciences

## Abstract

**Purpose:**

To present the translation into Brazilian Portuguese and cross-cultural adaptation of the *Apraxia of Speech Rating Scale (ASRS)* version 3.5.

**Methods:**

Validation study restricted to translation and cross-cultural adaptation. The following steps were carried out: translation and synthesis of translations; verification of applicability of the scale synthesis by judges recruited for this purpose; analysis of the relevance and feasibility of the scale calculated by the Content Validity Index (CVI), individual (CVI-I) and total (CVI-T). Eighteen speech therapists were selected. Their answers were used for the analysis of agreement (intraclass correlation coefficients - ICC) and for the calculation of the Content Validity Index (CVI). Finally, the synthesis of the translation was matched in terms of semantic, idiomatic, experiential, conceptual, syntactic, grammatical, and operational equivalence.

**Results:**

The ICC ranged between 0.83 and 0.94. Six items obtained values higher than 0.9. The other items presented values between 0.8 and 0.9. The CVI-I and CVI-T had excellent values (CVI ≥ 0.78) for relevance and feasibility.

**Conclusion:**

The Brazilian version of the *ASRS* 3.*5* presents semantic, idiomatic, experiential, conceptual, and syntactic/grammatical equivalence to the original document. Thus, it is ready for the next validation steps.

## INTRODUCTION

Speech apraxia is a neurological speech disorder that affects the ability to plan or program specific motor commands to direct a speech sequence^(1-2)^. Speech planning and motor programming are differentiable pre-execution phases. Motor planning occurs in cortical motor areas in the dominant hemisphere, while motor programming is mediated by bilateral subcortical areas and cortical-subcortical circuits in the brain^(3)^. Reports of the motor control of human kinetics have focused on the role of specific areas or structures in the brain, such as the basal ganglia^(4-5)^, the cerebellum^(6-9)^, the supplementary motor area, the pre-motor, and the primary motor area^(10-12)^.

The diagnosis of apraxia of speech is challenging for the clinician because of its frequent co-occurrence with acquired language and speech disorders, such as aphasia and dysarthria. Speech articulation is impaired in different ways by these three disorders above, although errors in the production of speech sounds, which occur in these three conditions, may manifest in a similar way^(13)^.

Distortion errors, for example, are common to dysarthric and apraxic patients^(13)^. In turn, some manifestations are common in patients with non-fluent aphasia and patients with speech apraxia, such as difficulty initiating speech, restarts of phonemes or syllables, and audible or visible articulatory groping^(14)^. Effort characteristics during speech production, as well as sound/syllable repetitions and prolongations, can be found in apraxic, aphasic, and dysarthric individuals^(14)^. The only characteristics that have stood out as specific markers of speech apraxia are articulatory distortions, such as distorted substitutions or distorted additions^(14)^, and prosodic alterations^(3)^.

Researchers and clinicians have endeavored to develop clinical and instrumental assessment tools to perform a differential diagnosis of patients with acquired neurological disorders of speech resulting mainly from cerebrovascular accidents (CVAs) or neurodegenerative diseases^(15)^. One of the main reasons for this is because many cases of apraxia of speech are diagnosed as non-fluent aphasia since the latter is better known in the clinical environment by neurologists and speech therapists^(15)^.

Thus, a way to minimize confusion in the description and differential diagnosis is to identify and quantify the intensity of characteristics considered as consistent markers of a given diagnosis^(16)^. In this sense, the use of instruments with clear definitions of speech tasks and target characteristics that must be auditorily evaluated by the clinician, both in terms of typology and severity of errors, may improve the reliability and accuracy of the auditory-perceptual judgment in a differential diagnosis of speech disorders^(16)^.

In this perspective, the *Apraxia of Speech Rating Scale* (*ASRS*)^(17)^ seeks to assess the presence of speech apraxia and the frequency/severity of characteristics of this disorder, specifically for a differential diagnosis between aphasia, dysarthria, and speech apraxia. The first version of the *ASRS* aimed to assist in the description and quantification of 16 speech characteristics accepted by the clinical and scientific community as indicative of speech apraxia, evidencing a great potential for diagnosis in the clinical environment. In terms of reliability, the initial version of the *ASRS* obtained an inter-judge intraclass correlation coefficient (ICC) between 0.87 and 0.91, and an intra-judge ICC between 0.91 and 0.98. Furthermore, the agreement was above 90% for most items, so that only two items showed an agreement <90%^(17)^.

The *ASRS* does not replace the traditional clinical assessment of adult patients with speech and language disorders of neurological origin. It is a complementary tool to identify the presence and severity of speech apraxia in specific tasks. The main advantage of the *ASRS* is to gather the main perceptual characteristics related to apraxic individuals and establish possible overlaps of dysarthric and aphasic conditions. This allows establishing criteria for a differential diagnosis of apraxia and its severity, which may lead to improvements in therapeutic planning, prognosis, and monitoring of the effects of rehabilitation on the speech characteristics of apraxic patients in a qualitative and quantitative way.

Thus, the purpose of this article is to present the translation into Brazilian Portuguese and the cross-cultural adaptation of the *ASRS* 3.5. The *ASRS* 3.5 may be a useful and effective tool for a differential diagnosis and characterization of the severity of speech apraxia during speech therapy intervention.

## METHODS

This is an instrument validation study restricted to the stages of translation and cross-cultural adaptation. It was approved by the Ethics Committee in Research with Human Beings of the Health Sciences Center of its home institution under opinion no. 4,929,996 and CAAE no. 42985821.0. 0000.5188. It complies with the Resolution no. 466/2012 of the Brazilian National Health Council (CNS).All study participants signed the Informed Consent (IC), with collection of data in a virtual environment. Initially, Brazilian researchers contacted the authors of the ASRS 3.5 and obtained authorization for the scale validation process in Brazil.

The process of translating and adapting the *ASRS* 3.5 to Brazilian Portuguese followed the guidelines and recommendations for development and evidence of validity based on the content of the test proposed in the literature^(18)^, according to the following steps:

1. *Translation:* Translation of the original version into Brazilian Portuguese by two speech therapists who are native speakers of Brazilian Portuguese and fluent in the English language and culture, independently; one was a specialist in the area of Language and the other was a non-specialist. Speech therapists did not receive previous training or knowledge related to the aforementioned scale and were aware of the research objective. The versions of the scale were named T1 and T2.
2. *Synthesis of translations:* A committee formed by two research speech therapists involved in this project was created to compare the translated versions. This session had an expected average duration of 90 minutes and took place in a virtual platform moderated by one of the researchers who composed the committee. The final version was compiled and called T3.
3. *Stage of verification of applicability of synthesis of translations:* The recruitment of new judges for the stage of applicability of the synthesis of translations was announced. The objective of this step was to verify the understanding of items by the target population that will use the scale and possible operational difficulties related to its application. Considering that the *ASRS* 3.5 is a tool to be used by clinicians evaluating the patient's speech samples, the target population of this study consisted of speech therapists with experience in the treatment of patients with speech disorders of neurological origin.

For the recruitment of volunteer speech therapists for this stage, the research was publicized on the social media of the study laboratory. Interested parties accessed a *link* to the digital electronic form. The sample used for the analysis of agreement and the calculation of the Content Validity Index (CVI) was composed of 18 speech therapists who obtained a minimum score of five points without adaptation of the scoring system “The Fehring Model”^(19)^, which was prepared for this research. Regarding the academic level, bachelors in Speech-Language Pathology and Audiology was mentioned 14 (77.8%) times; clinical practice of at least five years in the field of neurological disorders of speech was mentioned ten (55.6%) times; and two (11.1%) respondents are researchers and professors, and published scientific articles in the area.

Initially, the participants had to read the informed consent and, if they were interested and available to participate in the research, they had to sign it electronically and proceed with answering a brief questionnaire. The T3 version was presented in the form, and volunteers judged the relevance of each item for the proposed objective in the scale, the viability of the item in the clinical evaluation of speech in the Brazilian cultural context, the operational transformations in items, and the adequacy of the T3 version.

Regarding relevance, the judges used a four-point *Likert* scale if they considered items (1) irrelevant, (2) little relevant, (3) relevant, or (4) very relevant. Regarding the feasibility of items, the judges should have marked them as (1) not feasible, (2) not very feasible, (3) feasible, or (4) very feasible. In addition, the judges were asked to justify the answers “not feasible” and “not very feasible” and forward suggestions for modification or comments that could be relevant. Finally, experts judged whether the synthesis of translations was adequate or inadequate for the Brazilian cultural context.

To analyze the relevance and feasibility of items, the calculation of the total CVI (CVI-T) and individual CVI (CVI-I) was performed^(20)^. The CVI was used to assess the percentage of judges who were in agreement about the aspect evaluated in the instrument. It allows both an analysis of each item individually and an analysis of the instrument as a whole.

To calculate the CVI-I, the evaluators' scores were taken into account as for relevance and feasibility of items. Scores varied from 1 to 4, as previously described. The CVI-I was calculated using the following formula:


$$IVC-I=\frac{número\;de\;respostas\;"3"\;ou\;"4"}{número\;total\;de\;respostas}$$ (1)

The CVI-T was calculated using a simple mean of all CVI-I obtained for items regarding relevance and feasibility, respectively. In this research, the following reference values^(20)^ were considered: excellent (CVI ≥ 0.78), good (0.60 ≥ CVI ≤ 0.77), and poor (CVI ≤ 0.59). Items with a value lower than 0.60 regarding relevance or feasibility were mandatorily reanalyzed by the researchers and reformulated in the *ASRS*. As for the CVI-T, the arithmetic mean of all CVI-I scores in the four aspects considered was considered, with a minimum acceptable value of 0.90^(20)^.

The result of the CVI and the suggestions of the expert committee were evaluated by the researchers. The judges pondered on whether or not to modify the translation/adaptation of specific items.

1. *Back-translation:* The new version of the instrument (T4) was sent to an English-speaking professional, whose native language is English, but fluent in Brazilian Portuguese, without prior knowledge of the *ASRS* 3.5, who performed its back-translation. At the end of this stage, the scale was called *ASRS* 3.5 - back-translated version (T5).
2. *Final synthesis:* Two researchers responsible for this research evaluated whether the T5 version is compatible with the original version of the scale, specifically in terms of semantic, idiomatic, experiential, conceptual, syntactic/grammatical, and operational equivalences^(21)^. Equivalences were assessed by consensus between the two researchers.

Regarding semantic equivalence, it was verified whether the words have the same meaning. The language equivalence corresponds to the need or not to formulate an expression equivalent to colloquial idiomatic expressions that are difficult to translate. Experiential equivalence is related to the need or not to replace the original item with a similar item that exists in the target culture. In conceptual equivalence, the evaluators identified whether there are words or expressions that have a different conceptual meaning between cultures that could justify the replacement of such word or expression. As for cultural equivalence, the judges made necessary orthographic or grammatical adjustments to the scale items. Finally, the judgment of operational equivalence was carried out, assessing whether the procedures of the application of the *ASRS* 3.5 needed to be modified for application^(21)^. At the end of this step, the final version of the *ASRS* 3.5 was obtained.

In addition, the Intraclass Correlation Coefficient (ICC) was obtained. The ICC is a coefficient of agreement widely used to measure the reliability of measurements when comparing two or more evaluators.

The interpretation of the magnitude of the ICC is as 0 (absence), 0-0.19 (poor), 0.20-0.39 (weak), 0.30-0.59 (moderate), 0.60-0 .79 (substantial), and ≥ 0.80 (excellent).

## RESULTS

The final version of the *ASRS 3.5* was translated into Brazilian Portuguese and cross-culturally adapted (Appendix A). Chart 1 shows each stage of the translation and cross-cultural adaptation process.

**Chart 1:** Versions obtained during the process of translation into Brazilian Portuguese and cross-cultural adaptation of the Apraxia of Speech Rating Scale 3.5

Original	Synthesis of translations 1 (T1)	Synthesis of translations 2 (T2)	Version T3 (Synthesis of T1+T2)	Retranslated Version	Final Version
1- Sound distortions (excluding distorted substitutions or distorted additions)	Distorções de som (excluindo substituições distorcidas ou adições distorcidas)	Distorções de som (excluindo substituições distorcidas ou adições distorcidas)	Distorções de som (excluindo substituições distorcidas ou adições distorcidas)	Sound distortions (excluding distorted substitutions or distorted additions	Distorções de som (excluindo substituições distorcidas ou adições distorcidas)
2- Distorted sound substitutions	Substituições de som distorcido	Substituições de som distorcido	Substituições de som distorcido	Distorted sound substitutions	Substituições de som distorcido
3- Distorted sound additions (including intrusive schwa)	Adições de som distorcido (incluindo schwa intrusivo)	Adições de som distorcido (incluindo vogal intrusiva)	Adições de som distorcido (incluindo vogal intrusiva)	Distorted sound additions (including intrusive schwa)	Adições de som distorcido (incluindo vogal intrusiva)
4- Increased sound distortions or distorted sound substitutions with increased utterance length or increased syllable/word articulatory complexity	Aumento das distorções de som ou substituições de som distorcidas com aumento do comprimento do enunciado ou aumento da complexidade articulatória de sílaba / palavra	Aumento das distorções de som ou substituições de som distorcidas com aumento do tamanho do enunciado ou aumento da complexidade articulatória de sílaba / palavra	Aumento das distorções de som ou substituições de som distorcidas com aumento do tamanho do enunciado ou aumento da complexidade articulatória de sílaba / palavra	Increased sound distortions or distorted sound substitutions with increased utterance length or increased syllable/word articulatory complexity	Aumento das distorções de som ou substituições de som distorcidas com aumento do tamanho do enunciado ou aumento da complexidade articulatória de sílaba / palavra
5- Syllable segmentation within words > 1 syllable (Brief silent interval between syllables and/or inappropriate equalized stress across syllables)	Segmentação de sílaba dentro de palavras > 1 sílaba (breve intervalo silencioso entre as sílabas e / ou tonicidade inadequada entre as sílabas)	Segmentação de sílaba dentro de palavras > 1 sílaba (breve intervalo silencioso entre as sílabas e / ou tonicidade inadequada entre as sílabas)	Segmentação de sílaba dentro de palavras > 1 sílaba (breve intervalo silencioso entre as sílabas e / ou tonicidade inadequada entre as sílabas)	Syllable segmentation within words > 1 syllable (Brief silent interval between syllables and/or inappropriate equalized stress across syllable)	Segmentação de sílaba dentro de palavras > 1 sílaba (breve intervalo silencioso entre as sílabas e / ou tonicidade inadequada entre as sílabas)
6- Syllable segmentation across words in phrases/sentences (Increased inter-word intervals and/or inappropriate equalized stress across words)	Segmentação de sílaba entre palavras em frases / sentenças (aumento dos intervalos entre palavras e / ou tonicidade inadequada entre palavras)	Segmentação de sílaba entre palavras em frases / sentenças (aumento dos intervalos entre palavras e / ou tonicidade inadequada entre palavras)	Segmentação de sílaba entre palavras em frases / sentenças (aumento dos intervalos entre palavras e / ou tonicidade inadequada entre palavras)	Syllable segmentation across words in phrases/sentences (Increased inter-word intervals and/or inappropriate equalized stress across words)	Segmentação de sílaba entre palavras em frases / sentenças (aumento dos intervalos entre palavras e / ou tonicidade inadequada entre palavras)
7- Slow overall speech rate (apart from pauses for word retrieval and/or verbal formulation)	Velocidade geral de fala lenta (exceto pausas para recuperação de palavras e / ou formulação verbal	Velocidade geral de fala lenta (exceto pausas para recuperação de palavras e / ou formulação verbal)	Velocidade geral de fala lenta (exceto pausas para recuperação de palavras e / ou formulação verbal	Slow overall speech rate (apart from pauses for word retrieval and/or verbal formulation)	Velocidade geral de fala lenta (exceto pausas para recuperação de palavras e / ou formulação verbal
8- Lengthened vowel &/or consonant segments independent of overall slow speaking rate	Vogais e / ou segmentos consonantais alongados, independentemente da velocidade geral de fala lenta	Vogais e / ou segmentos consonantais alongados, independentemente da velocidade geral de fala lenta	Vogais e / ou segmentos consonantais alongados, independentemente da velocidade geral de fala lenta quando fala	Lengthened vowel &/or consonant segments independent of overall slow speaking rate	Vogais e / ou segmentos consonantais alongados, independentemente da velocidade geral de fala lenta
9- RATE ONLY FOR AMRs (alternating motion rates, as in rapid repetition of “puh puh puh”): Slow and/or off-target (in place, manner, and/or voicing) 0= AMRs normal; 1= rare and mild, 2= frequent but mild; 3 = moderate, 4 = severe	AVALIAÇÃO SOMENTE PARA AMRs (avaliações de movimento alternado, como na repetição rápida de “pa pa pa”): Lento e / ou fora do alvo (no ponto, modo e/ou vozeamento) 0 = AMRs normais; 1 = raro e leve, 2 = frequente, mas leve; 3 = moderado, 4 = intenso	AVALIAÇÃO SOMENTE PARA TMFA (avaliações da tarefa motora de fala alternada, como na repetição rápida de “pa pa pa”): Lento e / ou fora do alvo (no ponto, modo e/ou vozeamento) 0 = TMFA normais; 1 = raro e leve, 2 = frequente, mas leve; 3 = moderado, 4 = intenso	AVALIAÇÃO SOMENTE PARA TMFA (avaliações da tarefa motora de fala alternada, como na repetição rápida de “pa pa pa”): Lento e / ou fora do alvo (no ponto, modo e/ou vozeamento) 0 = TMFA normais; 1 = raro e leve, 2 = frequente, mas leve; 3 = moderado, 4 = intenso	RATE ONLY FOR AMRs (alternating motion rates, as in rapid repetition of “puh puh puh”): Slow and/or off-target (in place, manner, and/or voicing) 0= AMRs normal; 1= rare and mild, 2= frequent but mild; 3 = moderate, 4 = severe	AVALIAÇÃO SOMENTE PARA TMFA (avaliações da tarefa motora de fala alternada, como na repetição rápida de “pa pa pa”): Lento e / ou fora do alvo (no ponto, modo e/ou vozeamento) 0 = TMFA normais; 1 = raro e leve, 2 = frequente, mas leve; 3 = moderado, 4 = intenso
10- RATE ONLY FOR SMRs (sequential motion rates, as in rapid repetition of “puh tuhkuh”): Slow (gaps within sequences), segmented (gaps between sequences), incorrectly sequenced, and/or off-target (in place, manner, and/or voicing) 0= SMRs normal; 1= any one of the listed features, 2= any two of the listed features; 3 = any three of the listed features, 4 = four of the listed features	AVALIAÇÃO SOMENTE PARA SMRs (avaliações de movimento sequencial, como na repetição rápida de “pa ta ka”): Lento (lacunas dentro das sequências), segmentado (lacunas entre as sequências), sequenciado incorretamente e / ou fora do alvo (no ponto, modo e/ou vozeamento) 0 = SMRs normal; 1 = qualquer um dos recursos listados, 2 = quaisquer dois dos recursos listados, 3 = quaisquer três dos recursos listados, 4 = quatro dos recursos listados	AVALIAÇÃO SOMENTE PARA TMFS (avaliações da tarefa motora de fala sequenciada, como na repetição rápida de “pa ta ka”): Lento (intervalos dentro das sequências), segmentado (intervalos entre as sequências), sequenciado incorretamente e / ou fora do alvo (no ponto, modo e/ou vozeamento) 0 =TMFS normal; 1 = qualquer um dos recursos listados, 2 = quaisquer dois dos recursos listados, 3 = quaisquer três dos recursos listados, 4 = quatro dos recursos listados	AVALIAÇÃO SOMENTE PARA TMFS (avaliações da tarefa motora de fala sequenciada, como na repetição rápida de “pa ta ka”): Lento (intervalos dentro das sequências), segmentado (intervalos entre as sequências), sequenciado incorretamente e / ou fora do alvo (no ponto, modo e/ou vozeamento) 0 =TMFS normal; 1 = qualquer um dos recursos listados, 2 = quaisquer dois dos recursos listados, 3 = quaisquer três dos recursos listados, 4 = quatro dos recursos listados	RATE ONLY FOR SMRs (sequential motion rates, as in rapid repetition of “puh tuhkuh”): Slow (gaps within sequences), segmented (gaps between sequences), incorrectly sequenced, and/or off-target (in place, manner, and/or voicing) 0= SMRs normal; 1= any one of the listed features, 2= any two of the listed features; 3 = any three of the listed features, 4 = four of the listed features	AVALIAÇÃO SOMENTE PARA TMFS (avaliações da tarefa motora de fala sequenciada, como na repetição rápida de “pa ta ka”): Lento (intervalos dentro das sequências), segmentado (intervalos entre as sequências), sequenciado incorretamente e / ou fora do alvo (no ponto, modo e/ou vozeamento) 0 =TMFS normal; 1 = qualquer um dos recursos listados, 2 = quaisquer dois dos recursos listados, 3 = quaisquer três dos recursos listados, 4 = quatro dos recursos listados
11- One or both of the following: Consistently reduced words per breath group during phrase/sentence production relative to maximum vowel duration; reduced # of AMR repetitions per breath group in the absence of decreased respiratory capacity. Score on average number of syllables/repetitions per breath group across tasks: 0 = more than 7; 1= 6-7; 2 = 4-5; 3 = 3-4; 4 = 2 orless	Um ou ambos dos seguintes: Palavras consistentemente reduzidas por grupo de respiração durante a produção de frase / frase em relação à duração máxima da vogal; número reduzido de repetições de AMR por grupo de respiração na ausência de capacidade respiratória diminuída. Pontuação no número médio de sílabas / repetições por grupo de respiração nas tarefas: 0 = mais de 7; 1 = 6-7; 2 = 4-5; 3 = 3-4; 4 = 2 ou menos	Um ou ambos dos seguintes: Palavras consistentemente reduzidas por grupo respiratório durante a produção de frase / frase em relação à duração máxima da vogal; número reduzido de repetições de TMFS por grupo respiratório, na ausência de capacidade respiratória diminuída. Pontuação no número médio de sílabas / repetições por grupo de respiração nas tarefas: 0 = mais de 7; 1 = 6-7; 2 = 4-5; 3 = 3-4; 4 = 2 ou menos	Um ou ambos dos seguintes: Palavras consistentemente reduzidas por grupo respiratório durante a produção de frase / frase em relação à duração máxima da vogal; número reduzido de repetições de TMFS por grupo respiratório, na ausência de capacidade respiratória diminuída. Pontuação no número médio de sílabas / repetições por grupo de respiração nas tarefas: 0 = mais de 7; 1 = 6-7; 2 = 4-5; 3 = 3-4; 4 = 2 ou menos	One or both of the following: Consistently reduced words per breath group during phrase/sentence production relative to maximum vowel duration; reduced # of AMR repetitions per breath group in the absence of decreased respiratory capacity. Score on average number of syllables/repetitions per breath group across tasks: 0 = more than 7; 1= 6-7; 2 = 4-5; 3 = 3-4; 4 = 2 orless	Um ou ambos dos seguintes: Palavras consistentemente reduzidas por grupo respiratório durante a produção de frase / frase em relação à duração máxima da vogal; número reduzido de repetições de TMFS por grupo respiratório, na ausência de capacidade respiratória diminuída. Pontuação no número médio de sílabas / repetições por grupo de respiração nas tarefas: 0 = mais de 7; 1 = 6-7; 2 = 4-5; 3 = 3-4; 4 = 2 ou menos
12- Silent articulatory groping	Silêncio articulatório nas tentativas	Silêncio articulatório nas tentativas	Silêncio articulatório nas tentativas	Silent articulatory groping	Silêncio articulatório nas tentativas
13- Audible false starts/restarts or groping including sound repetitions, excluding fillers and unambiguous semantic false starts (e.g., spoo…fork)	Inícios / reinícios falsos audíveis ou nas tentativas incluindo repetições de som, excluindo enchimentos e falsos inícios semânticos inequívocos (ex.: colheee... garfo)	Inícios / reinícios falsos audíveis ou nas tentativas incluindo repetições de som, excluindo pausas preenchidas e falsos inícios semânticos inequívocos (ex.: colheee... garfo)	Inícios / reinícios falsos audíveis ou nas tentativas incluindo repetições de som, excluindo pausas preenchidas e falsos inícios semânticos inequívocos (ex.: colheee... garfo)	Audible false starts/restarts or groping including sound repetitions, excluding fillers and unambiguous semantic false starts (e.g., spoo…fork)	Inícios / reinícios falsos audíveis ou nas tentativas incluindo repetições de som, excluindo pausas preenchidas e falsos inícios semânticos inequívocos (ex.: colheee... garfo)

The intraclass correlation coefficients (Table 1) ranged between 0.83 and 0.94. The items 1, 5, 6, 9, 10 and 12 obtained values higher than 0.9. The other items presented values between 0.8 and 0.9. Thus, the high value of the ICC for the analysis of items suggests that the variability between the evaluators' responses was low. This is a positive result for agreement analysis.

**Table 1:** Analysis of the Interclass Correlation Coefficient - ICC of items of Apraxia of Speech Rating Scale 3.5 translated into Brazilian Portuguese and cross-cultural adaptation

ITEM	ICC	Confidence Interval - 95%	
		Lower limit	Upper limit
Sound distortions (excluding distorted substitutions or distorted additions)	0.91	0.71	0.99
Distorted sound substitutions	0.88	0.64	0.99
Distorted sound additions (including intrusive schwa)	0.83	0.53	0.99
Increased sound distortions or distorted sound substitutions with increased utterance length or increased syllable/word articulatory complexity	0.87	0.62	0.99
Syllable segmentation within words > 1 syllable (Brief silent interval between syllables and/or inappropriate equalized stress across syllables)	0.94	0.80	0.99
Syllable segmentation across words in phrases/sentences (Increased inter-word intervals and/or inappropriate equalized stress across words)	0.90	0.69	0.99
Slow overall speech rate (apart from pauses for word retrieval and/or verbal formulation)	0.84	0.57	0.99
Lengthened vowel &/or consonant segments independent of overall slow speaking rate	0.86	0.59	0.99
RATE ONLY FOR AMRs (alternating motion rates, as in rapid repetition of “puh puh puh”): Slow and/or off-target (in place, manner, and/or voicing) 0= AMRs normal; 1= rare and mild, 2= frequent but mild; 3 = moderate, 4 = severe	0.92	0.74	0.99
RATE ONLY FOR SMRs (sequential motion rates, as in rapid repetition of “puh tuh kuh”): Slow (gaps within sequences), segmented (gaps between sequences), incorrectly sequenced, and/or off-target (in place, manner, and/or voicing) 0= SMRs normal; 1= any one of the listed features, 2= any two of the listed features; 3 = any three of the listed features, 4 = four of the listed features	0.93	0.75	0.99
One or both of the following: Consistently reduced words per breath group during phrase/sentence production relative to maximum vowel duration; reduced # of AMR repetitions per breath group in the absence of decreased respiratory capacity. Score on average number of syllables/repetitions per breath group across tasks: 0 = more than 7; 1= 6-7; 2 = 4-5; 3 = 3-4; 4 = 2 or less	0.87	0.62	0.99
Silent articulatory groping	0.91	0.70	0.99
Audible false starts/restarts or groping including sound repetitions, excluding fillers and unambiguous semantic false starts (e.g., spoo…fork)	0.88	0.64	0.99
**Total**	**0.85**	**0.78**	**0.90**

Table 2 shows the results of the analysis by CVI-I and CVI-T regarding the relevance and feasibility of the items.

**Table 2:** Analysis of Content Validation Index - CVI of items of Apraxia of Speech Rating Scale 3.5 translated into Brazilian Portuguese and cross-cultural adaptation

ITEM	CVI-I	
	Relevance	Feasibility
Sound distortions (excluding distorted substitutions or distorted additions)	1.000	0.944
Distorted sound substitutions	1.000	0.889
Distorted sound additions (including intrusive schwa)	0.889	0.833
Increased sound distortions or distorted sound substitutions with increased utterance length or increased syllable/word articulatory complexity	0.944	0.944
Syllable segmentation within words > 1 syllable (Brief silent interval between syllables and/or inappropriate equalized stress across syllables)	1.000	1.000
Syllable segmentation across words in phrases/sentences (Increased inter-word intervals and/or inappropriate equalized stress across words)	0.944	0.944
Slow overall speech rate (apart from pauses for word retrieval and/or verbal formulation)	0.944	0.944
Lengthened vowel &/or consonant segments independent of overall slow speaking rate	0.944	0.889
RATE ONLY FOR AMRs (alternating motion rates, as in rapid repetition of “puh puh puh”): Slow and/or off-target (in place, manner, and/or voicing) 0= AMRs normal; 1= rare and mild, 2= frequent but mild; 3 = moderate, 4 = severe	1.000	1.000
RATE ONLY FOR SMRs (sequential motion rates, as in rapid repetition of “puh tuh kuh”): Slow (gaps within sequences), segmented (gaps between sequences), incorrectly sequenced, and/or off-target (in place, manner, and/or voicing) 0= SMRs normal; 1= any one of the listed features, 2= any two of the listed features; 3 = any three of the listed features, 4 = four of the listed features	1.000	1.000
One or both of the following: Consistently reduced words per breath group during phrase/sentence production relative to maximum vowel duration; reduced # of AMR repetitions per breath group in the absence of decreased respiratory capacity. Score on average number of syllables/repetitions per breath group across tasks: 0 = more than 7; 1= 6-7; 2 = 4-5; 3 = 3-4; 4 = 2 or less	0.944	0.944
Silent articulatory groping	1.000	1.000
Audible false starts/restarts or groping including sound repetitions, excluding fillers and unambiguous semantic false starts (e.g., spoo…fork)	1.000	0.944
**Total**	**0.97**	**0.94**

The coefficients calculated for relevance obtained values equal to or higher than 0.89, while for feasibility coefficients obtained values equal to or higher than 0.83 (Item 3). Thus, the values calculated for the CVI suggest excellent parameters for the analysis of relevance and feasibility.

## DISCUSSION

The methodology used in this study enabled the translation into Brazilian Portuguese and the cross-cultural adaptation of the assessment scale *Apraxia of Speech Scale* 3.5 (*ASRS*). The *ASRS* 3.5^(17)^ is a clinical assessment scale of acquired speech apraxia with adequate psychometric properties and, with the completion of the validation process, it will be able to contribute to the identification of the presence of speech apraxia, classify the frequency/severity of speech manifestations, and provide a differential diagnosis between aphasia, acquired speech apraxia, and dysarthria.

For the original validation of the *ASRS* 3.5^(17)^, the evaluation of three experienced clinicians was a reference standard. They had access to the speech tasks of 133 patients previously diagnosed with speech apraxia and/or aphasia, including answers to questions, repetition of words and phrases, naming, sentence-filling tasks, spontaneous speech, alternating speech motor task, and sequenced speech motor task. Based on the perceptual judgment of these tasks, the evaluators established the diagnosis of aphasia (under-specifying its typology) or speech apraxia and the severity of the manifestations^(17)^.

Subsequently, two other clinicians confirmed this diagnosis by consensus. They were not involved in the process and had access to speech samples. The results of the *ASRS* 3.5 were then compared with the results of the assessment cited above. Patients with speech apraxia received higher scores than non-apraxic patients, establishing a cut-off value of eight points. The sensitivity was 96% and the specificity was 100% in the identification of apraxia. There was a strong positive correlation (r=0.88) between the *ASRS* 3.5 score and the severity previously indicated by clinicians in the reference standard, which reinforces the potential of the scale to establish the severity of the apraxic condition^(17)^.

Thus, there was a potential for using the *ASRS* 3.5 in the clinical environment to identify and quantify speech apraxia in general. It is expected, therefore, that it is possible to observe this potential in Brazilian Portuguese with the continuity of the validation study that began in this study. The use of the *ASRS* 3.5 scale leads to benefits for professionals who work with patients with acquired neurological disorders, favoring an early identification of the speech disorder, the typology and severity of characteristics, and the selection of a more assertive speech therapy treatment.

Thus, the process of translation, cross-cultural adaptation, and back-translation of the *ASRS* 3.5 into Brazilian Portuguese maintained the veracity of the information conveyed by the original version. It is suggested that, with this process, the linguistic and cultural discrepancies are solved, as it is essential that the population context is considered at the moment of translation^(22-23)^.

During translation and synthesis, there was a search for appropriate words and phrases to the cultural context of Brazilian Portuguese and for the maintenance of the original meaning of the scale. Thus, the importance of the expert committee to ensure the cultural and linguistic equivalence of the *ASRS* 3.5 is highlighted. In this sense, the adequate values of CVI-I and CVI-Q reinforced the relevance and feasibility of the scale for the Brazilian culture.

The item about “sound distortions” presented relevance, feasibility, and adequacy with excellent parameters; its CCI value was considered high (0.91). Sound distortion is a phonetic alteration that does not involve the phonological rules of the language. It is characterized by motor difficulties in the production of sounds, such as imprecision of location, time, pressure, and speed, resulting in a non-standard speech sound^(24)^.

The item “Distorted sound substitutions” was deemed relevant, feasible, and adequate. Distorted sound substitutions are frequent manifestations of apraxia of speech. This is because the apraxic individual replaces the sounds that he or she previously distorted, thus presenting inconsistent errors in the speech flow^(2,25)^.

The item “Increased sound distortions or distorted sound substitutions with increased utterance length or increased syllable/word articulatory complexity” was considered relevant, feasible, and adequate. Apraxic individuals may present more difficulties with increasing articulatory complexity^(25)^.

The “syllable segmentation within words” is a characteristic that reflects a difficulty in the co-articulation of syllables and frequent segmentation, which may result in prosodic flaws. This item can be quantified, and the rate and duration of word and sentence productions can be valuable in diagnosis, with a high predictive value of speech apraxia^(26)^.

The items “Syllable segmentation across words in phrases/sentences (Increased inter-word intervals and/or inappropriate equalized stress across words),” “slow overall speech rate,” and “Lengthened vowel &/or consonant segments independent of overall slow speaking rate” were judged relevant, feasible, and appropriate. Apraxia affects the production of speech sounds and their organization in the formation of syllables and words^(1)^. In this way, the apraxic individual may present a slower and segmented speech, as failures in motor planning make it difficult to link the syllables and words and gaps in patterns of intonation, rhythm, and melody of speech^(27)^.

The item related to “rate only for the alternating motion rates and sequential motion rates” was judged as feasible, relevant, and adequate. These two tasks are important for the diagnosis of apraxia, as they are used to emit speech segments in relation to the parameters of speed, intensity, rhythm, precision, duration of emission, and coefficients of variation, parameters that are evaluated and usually altered in apraxia^(3,26)^ due to difficulties in pneumo-phono-articulatory coordination and in the motor programming of muscle programs related to breathing and speech^(28)^.

Therefore, the item related to “Consistently reduced words per breath group during phrase/sentence production relative to maximum vowel duration; reduced # of sequenced speech motor task repetitions per breath group in the absence of decreased respiratory capacity” was also judged relevant and feasible.

The item “articulatory silence in attempts” was considered relevant, feasible, and adequate. Apraxic patients struggle to find the right joint posture. Facial mimics are usually surrounded by silent movements of the lips in a contorted and forced manner, presenting an articulatory groping and productive effort^(29)^.

The item “audible false starts/restarts or groping including sound repetitions, excluding fillers and unambiguous semantic false starts ” was judged relevant, feasible, and adequate. This characteristic occurs due to an interruption of the motor programming, incorporating errors in motor commands that result in restarts and initial repetitions of syllables^(30)^.

In short, the values calculated by the CCI and CVI had excellent parameters in terms of adequation, relevance, and feasibility. The validation process of the *ASRS* 3.5 scale will continue from the translated and adapted version**.**

## CONCLUSION

The Brazilian version of the *Apraxia of Speech Rating Scale 3.5* presents semantic, idiomatic, experiential, conceptual, and syntactic/grammatical equivalence with the original version. Thus, it is ready for the next validation steps.

## Appendix A “*Escala de Avaliação da Apraxia de Fala*”. Translated into Brazilian Portuguese and cross-culturally adapted version of the Apraxia of Speech Rating Scale 3.5

***Escala de Avaliação da Apraxia de Fala***

**-*Apraxia of Speech Rating Scale 3.5* -**

**Nome completo: ______________________________________________________ _________ Idade: _____________**

**Data de nascimento: ___/___/___ Data da avaliação: ___/___/___ Examinador: ___________________________________**

**Table:**

**ESCORE**	**0**	**1**	**2**	**3**	**4**
**DESCRIÇÃO**	**Não observado em nenhuma tarefa**	**Raro**	**Frequente, mas não pervasivo**	**Ocorre quase sempre, em grau menos severo**	**Ocorre quase sempre, em grau mais severo**
**DIRETRIZES**	Menos de uma ocorrência	Mais de uma ocorrência, mas em menos de 20% das palavras	Observado em cerca de 20-50% das palavras	Observado na maioria das palavras	Observado em quase todas as palavras
**EXCEÇÕES**				Pontuação não superior a “2” se presente apenas durante a repetição	Pontuação “4” se a inteligibilidade for mais do que ligeiramente reduzida
Desempenho na TMFA e na TMFS considerados para os itens 9-11					
CARACTERÍSTICAS FONÉTICAS					
**1** ^APRAXIA/DISARTRIA^	Distorções de som (excluindo substituições distorcidas ou adições distorcidas)				
**2** ^APRAXIA^	Substituições de som distorcido				
**3** ^APRAXIA^	Adições de som distorcido (incluindo vogal intrusiva)				
**4** ^APRAXIA^	Aumento das distorções de som ou substituições de sons distorcidos com aumento do tamanho do enunciado ou aumento da complexidade articulatória de sílaba / palavra				
CARACTERÍSTICAS PROSÓDICAS					
**5** ^APRAXIA/DISARTRIA^	Segmentação de sílaba dentro de palavras > 1 sílaba (breve intervalo silencioso entre as sílabas e / ou tonicidade inadequada entre as sílabas)				
**6** ^APRAXIA/DISARTRIA^	Segmentação de sílaba entre palavras em frases / sentenças (aumento dos intervalos entre palavras e / ou tonicidade inadequada entre palavras)				
**7** ^APRAXIA/DISARTRIA^	Velocidade geral de fala lenta (exceto pausas para recuperação de palavras e / ou formulação verbal)				
**8** ^APRAXIA/DISARTRIA^	Vogais e / ou segmentos consonantais alongados, independentemente da velocidade geral de fala lenta				
OUTROS					
**9** ^APRAXIA/DISARTRIA/AFASIA^	AVALIAÇÃO SOMENTE PARA TMFA (avaliações da tarefa motora de fala alternada, como na repetição rápida de “pa pa pa”): Lento e / ou fora do alvo (no ponto, modo e/ou vozeamento) 0 = TMFA normais; 1 = raro e leve, 2 = frequente, mas leve; 3 = moderado, 4 = intenso				
**10** ^APRAXIA/DISARTRIA/AFASIA^	AVALIAÇÃO SOMENTE PARA TMFS (avaliações da tarefa motora de fala sequenciada, como na repetição rápida de “pa ta ka”): Lento (intervalos dentro das sequências), segmentado (intervalos entre as sequências), sequenciado incorretamente e / ou fora do alvo (no ponto, modo e/ou vozeamento) 0 =TMFS normal; 1 = qualquer um dos recursos listados, 2 = quaisquer dois dos recursos listados, 3 = quaisquer três dos recursos listados, 4 = quatro dos recursos listados				
**11** ^APRAXIA^	Um ou ambos dos seguintes: Palavras consistentemente reduzidas por grupo respiratório durante a produção de frase / frase em relação à duração máxima da vogal; número reduzido de repetições de TMFS por grupo respiratório, na ausência de capacidade respiratória diminuída. Pontuação no número médio de sílabas / repetições por grupo de respiração nas tarefas: 0 = mais de 7; 1 = 6-7; 2 = 4-5; 3 = 3-4; 4 = 2 ou menos				
**12** ^APRAXIA/AFASIA^	Silêncio articulatório nas tentativas				
**13** ^APRAXIA/DISARTRIA/AFASIA^	Inícios / reinícios falsos audíveis ou nas tentativas incluindo repetições de som, excluindo pausas preenchidas e falsos inícios semânticos inequívocos (ex.: colheee... garfo)				
ESCORE TOTAL					

**Caption:** TMFA = Alternating motion rates; TMFS = Sequential motion rates; APRAXIA = Primary distinguishing features (rare overlap with dysarthria or aphasia); APRAXIA/DISARTRIA = Distinguishing features unless dysarthria present; APRAXIA/AFASIA = Distinguishing features unless aphasia present; APRAXIA/DISARTRIA/AFASIA = Distinguishing feature unless aphasia and/or dysarthria are present

Speech tasks indicated for the assessment: Spontaneous Speech, Conversation, Image Description/Oral Narrative Discourse, Repetition of words and phrases, Alternating motion rates and Sequential motion rates
