# Supplementary material for: Translation into Brazilian Portuguese and transcultural adaptation of the Apraxia of Speech Rating Scale 3.5
Source: Codas. 2023 Jun 30;35(3):e20220012. doi: 10.1590/2317-1782/20232022012en (PMC10348665; doi:10.1590/2317-1782/20232022012en)
Supplement: Translation

# Translation: Tradução e adaptação transcultural do *Apraxia of Speech Rating Scale* 3.5 para o português brasileiro

**DOI:** 10.1590/2317-1782/20232022012pt

**Authors:** Dhébora Heloísa Nascimento dos Santos, Ivonaldo Leidson Barbosa Lima, Leonardo Wanderley Lopes

**Affiliations:** 1 Programa Associado de Pós-graduação em Fonoaudiologia, Universidade Federal da Paraíba - UFPB - João Pessoa (PB), Brasil.; 2 Departamento de Fonoaudiologia, Universidade Federal do Rio Grande do Norte - UFRN - Natal (RN), Brasil.; 3 Departamento de Fonoaudiologia, Universidade Federal da Paraíba - UFPB - João Pessoa (PB), Brasil.

**Keywords:** Apraxias, Distúrbios da Fala, Diagnóstico Diferencial, Estudo de Validação, Psicometria, Fonoaudiologia

## Abstract

**Objetivo:**

Apresentar a tradução e adaptação transcultural do *Apraxia of Speech Rating Scale (ASRS)* versão 3.5 para o português brasileiro.

**Método:**

Estudo de validação restrito à tradução e adaptação transcultural. Foram realizadas as seguintes etapas: tradução e síntese das traduções; verificação da aplicabilidade da síntese da escala por juízes, recrutados para tal finalidade; análise da relevância e da viabilidade da escala, calculadas pelo Índice de Validade de Conteúdo (IVC) individual (IVC-I) e total (IVC-T). Foram selecionados 18 fonoaudiólogos, cujas respostas foram utilizadas para a análise de concordância (coeficientes de correlação intraclasse - CCI) e o cálculo do Índice de Validade de Conteúdo (IVC). Por fim, a síntese da tradução foi equiparada quanto à equivalência semântica, idiomática, experiencial, conceitual, sintática, gramatical e operacional.

**Resultados:**

O CCI variou entre 0,83 e 0,94. Seis itens obtiveram valores superiores a 0,9. Os demais itens apresentaram valores entre 0,8 e 0,9. O IVC-I e IVC-T apresentaram excelentes valores (IVC ≥ 0,78) para a relevância e viabilidade.

**Conclusão:**

A versão brasileira do *ASRS 3.5* apresenta equivalência semântica, idiomática, experiencial, conceitual e sintática/gramatical em relação ao original, dessa forma, está apta para as próximas etapas de validação.

## INTRODUÇÃO

A apraxia de fala é um distúrbio neurológico da fala que afeta a capacidade de planejar ou programar comandos motores específicos para direcionar uma sequência da fala^(1-2)^. O planejamento e a programação motora da fala são fases de pré-execução diferenciáveis. O planejamento motor ocorre em áreas motoras corticais no hemisfério dominante, enquanto a programação motora é mediada por áreas subcorticais bilaterais e circuitos cortical-subcorticais no cérebro^(3)^. Relatos do controle motor da cinética humana enfocaram o papel de áreas ou estruturas específicas no cérebro, como os gânglios da base^(4-5)^, o cerebelo^(6-9)^, a área motora suplementar, a área pré-motora e a área motora primária^(10-12)^.

O diagnóstico da apraxia de fala é desafiador para o clínico devido à sua frequente coocorrência com transtornos adquiridos da linguagem e a da fala, como a afasia e a disartria. A fonoarticulação é prejudicada de formas distintas nesses três transtornos citados, embora os erros na produção dos sons da fala, que ocorrem nessas três condições, possam se manifestar de forma semelhante^(13)^.

Erros de distorções, por exemplo, são comuns aos pacientes disártricos e apráxicos^(13)^. Por sua vez, algumas manifestações são comuns a pacientes com afasia não fluente e a pacientes com apraxia de fala, como dificuldade de iniciar a fala, reinícios de fonemas ou sílabas e tateio articulatório audível ou visível^(14)^. As características de esforço durante a produção da fala, bem como as repetições e prolongamentos de som/sílaba podem ser encontradas em indivíduos apráxicos, afásicos e disártricos^(14)^. As únicas características que têm se destacado como marcadores específicos da apraxia de fala são as distorções articulatórias, como substituições distorcidas e adições distorcidas^(14)^, assim como as alterações prosódicas^(3)^.

Pesquisadores e clínicos têm se esforçado para desenvolver instrumentos de avaliação clínica e instrumental para realizar o diagnóstico diferencial de pacientes com alterações neurológicas de fala adquiridas, principalmente advindas de acidentes vasculares encefálicos (AVEs) ou de doenças neurodegenerativas^(15)^. Uma das principais motivações para isso é o fato de que muitos casos de apraxia de fala são diagnosticados como afasia não fluente, visto que esta última condição é mais conhecida no ambiente clínico por médicos neurologistas e fonoaudiólogos^(15)^.

Assim, uma das maneiras de minimizar confusões na descrição e diagnóstico diferencial é identificar e quantificar a intensidade das características consideradas como marcadores consistentes de um determinado diagnóstico^(16)^. Nesse sentido, o uso de instrumentos com definições claras das tarefas de fala e das características-alvo que devem ser avaliadas auditivamente pelo clínico, tanto em termos de tipologia quanto de severidade dos erros, pode melhorar a confiabilidade e a acurácia do julgamento perceptivo-auditivo no diagnóstico diferencial dos transtornos da fala^(16)^.

Nessa perspectiva, a *Apraxia of Speech Rating Scale* (*ASRS*)^(17)^ foi proposta com a finalidade de avaliar a presença da apraxia de fala e a frequência/severidade das características desse transtorno, especificamente para o diagnóstico diferencial entre afasia, disartria e apraxia de fala. A primeira versão da *ASRS* objetivou auxiliar na descrição e na quantificação de 16 características de fala aceitas pela comunidade clínica e científica como indicativas da apraxia de fala, demonstrando um grande potencial para diagnóstico no ambiente clínico. Em termos de confiabilidade, a versão inicial da *ASRS* obteve um coeficiente de correlação intraclasses (CCI) inter-juízes entre 0,87 e 0,91, assim como um CCI entre 0,91 e 0,98 intra-juízes. Além disso, observou-se concordância acima de 90% para a maioria dos itens, de modo que apenas dois itens apresentaram concordância <90%^(17)^.

A *ASRS* não substitui a avaliação clínica tradicional de pacientes adultos com transtornos de fala e linguagem de origem neurológica, mas constitui-se em uma ferramenta complementar para identificar a presença e a severidade da apraxia de fala a partir de tarefas específicas. A principal vantagem da *ASRS* é reunir as principais características perceptuais relacionadas aos indivíduos apráxicos, estabelecendo também as possíveis sobreposições dos quadros disártricos e afásicos. Isso permite estabelecer critérios para o diagnóstico diferencial da apraxia e da sua severidade, o que pode refletir em aprimoramentos no planejamento terapêutico, no prognóstico e no monitoramento do efeito da reabilitação nas características de fala do paciente apráxico de maneira quali e quantitativa.

Dessa forma, o objetivo deste artigo é apresentar a tradução e adaptação transcultural do *ASRS* 3.5 para o português brasileiro. O *ASRS* 3.5 pode ser uma ferramenta útil e efetiva para o diagnóstico diferencial e caracterização da severidade da apraxia de fala durante a intervenção fonoaudiológica.

## MÉTODO

Trata-se de um estudo de validação de instrumento, restrito as etapas de tradução e adaptação transcultural, aprovado pelo Comitê de Ética em Pesquisa com Seres Humanos do Centro de Ciências da Saúde da instituição de origem sob parecer 4.929.996 e CAAE 42985821.0.0000.5188, e está de acordo com a Resolução 466/2012, do Conselho Nacional de Saúde (CNS). Todos os participantes do estudo assinaram o Termo de Consentimento Livre e Esclarecido (TCLE), com coleta em ambiente virtual. Inicialmente, os pesquisadores brasileiros fizeram contato com os autores do ASRS 3.5 e receberam a autorização para o processo de validação da escala no Brasil.

O processo de tradução e adaptação do *ASRS* 3.5 para o português brasileiro foi norteado por diretrizes e recomendações de desenvolvimento e evidência de validade baseada no conteúdo do teste propostas na literatura^(18)^, conforme as etapas a seguir:

1. *Tradução:* tradução da versão original para o português brasileiro por dois fonoaudiólogos nativos do português brasileiro e fluentes no idioma e cultura inglesa, de forma independente, sendo um especialista na área de Linguagem e outro não especialista. Os fonoaudiólogos não receberam treinamento prévio ou conhecimento relacionado à referida escala e estavam cientes do objetivo da pesquisa. As versões da escala produzidas foram denominadas T1 e T2
2. *Síntese das traduções:* foi constituído um comitê formado por dois fonoaudiólogos pesquisadores envolvidos neste projeto comparando as versões traduzidas. Essa sessão teve duração média prevista de 90 minutos e ocorreu através de uma plataforma virtual, sob a moderação de um dos pesquisadores constituintes do comitê. A versão final foi concluída e chamada de T3.
3. *Etapa verificação de aplicabilidade da síntese das traduções:* foi divulgado o recrutamento de novos juízes para a etapa de aplicabilidade da síntese das traduções. O objetivo desta etapa foi a verificação da compreensão dos itens por parte da população-alvo que fará uso da escala e as possíveis dificuldades operacionais relacionadas à sua aplicação. Considerando que a *ASRS* 3.5 é uma ferramenta a ser utilizada pelo clínico ao avaliar as amostras de fala do paciente, a população-alvo deste estudo foi constituída por fonoaudiólogos com experiência no manejo de pacientes com transtornos da fala de origem neurológica.

Para o recrutamento de fonoaudiólogos voluntários para essa etapa, a pesquisa foi divulgada nas mídias sociais do laboratório do estudo. Os interessados acessaram um *link* para o formulário eletrônico digital. A amostra utilizada para a análise de concordância e o cálculo do Índice de Validade de Conteúdo (IVC) foi composta por 18 fonoaudiólogos que obtiveram escore mínimo de 5 ponto sem uma adaptação do sistema de pontuação “*The Fehring Model*”^(19)^ elaborada para esta pesquisa. Em relação ao nível acadêmico, a graduação em Fonoaudiologia foi citada 14 (77,8%) vezes; a prática clínica de pelo menos cinco anos na área dos transtornos neurológicos da fala foi citada 10 (55,6%) vezes; e 2 (11,1%) dos respondentes são pesquisadores, docentes e têm artigos científicos na área.

Inicialmente, os participantes tinham que ler o TCLE e, caso estivessem interessados e disponíveis em participar da pesquisa, deveriam assinar eletronicamente e proceder com a resposta a um breve questionário. No formulário, a versão da T3 foi apresentada e os voluntários julgaram a relevância de cada item para o objetivo proposto na escala, a viabilidade do item na avaliação clínica da fala no contexto cultural brasileiro, as transformações operacionais nos itens e a adequação da versão T3.

Quanto à relevância, os juízes marcaram em uma escala de *Likert* de quatro pontos se consideram (1) irrelevante, (2) pouco relevante, (3) relevante ou (4) muito relevante. Com relação à viabilidade dos itens, os juízes deverão marcar como (1) inviável, (2) pouco viável, (3) viável ou (4) muito viável. Além disso, foi solicitado que os juízes justificassem as respostas “inviável” e “pouco viável” e encaminhassem sugestões de modificação ou comentários que sejam pertinentes. Por fim, os especialistas julgaram se a síntese das traduções estava adequada ou inadequada ao contexto cultural brasileiro.

Para análise da relevância e viabilidade dos itens foi realizado o cálculo do IVC total (IVC-T) e individual (IVC-I)^(20)^. O IVC foi utilizado para avaliar a porcentagem de juízes que estavam em concordância sobre o aspecto avaliado no instrumento. Ele permite tanto uma análise de cada item individualmente, quanto a análise do instrumento como um todo.

Para o cálculo do IVC-I foram considerados os escores dos avaliadores quanto à relevância e viabilidade dos itens, que variavam de 1 a 4, conforme descrito anteriormente. O IVC-I foi calculado pela seguinte fórmula:


$$IVC-I=\frac{número\;de\;respostas\;"3"\;ou\;"4"}{número\;total\;de\;respostas}$$ (1)

O IVC-T foi calculado a partir da média simples de todos os IVC-Is obtidos nos itens quanto à relevância e viabilidade, respectivamente. Nesta pesquisa, foram considerados os seguintes valores de referência^(20)^: excelente (IVC ≥ 0,78), bom (0,60 ≥ IVC ≤ 0,77) e ruim (IVC ≤ 0,59). Os itens com valor inferior a 0,60 quanto à relevância ou viabilidades foram obrigatoriamente reanalisados pelos pesquisadores e reformulados no *ASRS*. Quanto ao IVC-T, foi considerada a média aritmética de todos os IVC-I nos quatro aspectos julgados, com valor mínimo aceitável de 0,90^(20)^.

O resultado do IVC e as sugestões do comitê de especialistas foram avaliados pelos pesquisadores responsáveis, os quais foram deliberados sobre a necessidade ou não de modificação na tradução/adaptação de itens específicos.

1. *Retrotradução:* a nova versão do instrumento (T4) foi enviada para um profissional habilitado em língua inglesa, cuja língua materna é inglês, mas com fluência no português brasileiro, sem conhecimento prévio da *ASRS* 3.5, que realizou sua retrotradução. Ao final dessa etapa, a escala foi denominada *ASRS* 3.5 - versão retrotraduzida (T5).
2. *Síntese final:* dois pesquisadores responsáveis pela presente pesquisa avaliaram se a versão T5 é compatível com a versão original da escala, especificamente quanto às equivalências semântica, idiomática, experiencial, conceitual, sintática/gramatical e operacional^(21)^. As equivalências foram avaliadas por consenso entre os dois pesquisadores.

Na equivalência semântica, foram observadas se as palavras têm o mesmo significado. A equivalência idiomática corresponde à necessidade ou não de formular uma expressão equivalente a expressões idiomáticas coloquiais difíceis de traduzir. A equivalência experiencial está relacionada a necessidade ou não de substituição do item original por um item semelhante que exista na cultura alvo. Na equivalência conceitual, os avaliadores identificaram se há palavras ou expressões que possuem significado conceitual diferente entre as culturas, o que justificaria a substituição de tal palavra ou expressão. Quanto à equivalência cultural, os juízes fizeram necessários ajustes ortográficos ou gramaticais nos itens da escala. Por fim, foi realizado o julgamento da equivalência operacional, avaliando se os procedimentos inerentes à aplicação da *ASRS* 3.5 precisavam ser modificados para a sua aplicação^(21)^. Ao término dessa etapa, foi obtida a versão final da *ASRS* 3.5.

Além disso, foi obtido o Coeficiente de Correlação Intraclasse (*Intraclass Correlation Coefficient* - CCI). O CCI trata-se de um coeficiente de concordância largamente utilizado para mensuração da confiabilidade de medidas quando se está fazendo comparação entre dois ou mais avaliadores.

A interpretação da magnitude do CCI é convencionada como: 0 (ausência), 0-0,19 (pobre), 0,20-0,39 (fraca), 0,30-0,59 (moderada), 0,60-0,79 (substancial), e ≥ 0,80 (excelente).

## RESULTADOS

A versão final do *ASRS 3.5* foi traduzida e adaptada transculturalmente para o português brasileiro (Apêndice A). Cada etapa do processo de tradução e adaptação transcultural estão inseridas no Quadro 1.

**Quadro1:** Versões obtidas ao longo do processo de tradução e adaptação transcultural do *Apraxia of Speech Rating Scale* 3.5 para o português brasileiro

Original	Síntese de Tradução 1 (T1)	Síntese de Tradução 2 (T2)	Versão T3 (Síntese de T1+T2)	Versão Retraduzida	Versão final
1- Sound distortions (excluding distorted substitutions or distorted additions)	Distorções de som (excluindo substituições distorcidas ou adições distorcidas)	Distorções de som (excluindo substituições distorcidas ou adições distorcidas)	Distorções de som (excluindo substituições distorcidas ou adições distorcidas)	Sound distortions (excluding distorted substitutions or distorted additions	Distorções de som (excluindo substituições distorcidas ou adições distorcidas)
2- Distorted sound substitutions	Substituições de som distorcido	Substituições de som distorcido	Substituições de som distorcido	Distorted sound substitutions	Substituições de som distorcido
3- Distorted sound additions (including intrusive schwa)	Adições de som distorcido (incluindo schwa intrusivo)	Adições de som distorcido (incluindo vogal intrusiva)	Adições de som distorcido (incluindo vogal intrusiva)	Distorted sound additions (including intrusive schwa)	Adições de som distorcido (incluindo vogal intrusiva)
4- Increased sound distortions or distorted sound substitutions with increased utterance length or increased syllable/word articulatory complexity	Aumento das distorções de som ou substituições de som distorcidas com aumento do comprimento do enunciado ou aumento da complexidade articulatória de sílaba / palavra	Aumento das distorções de som ou substituições de som distorcidas com aumento do tamanho do enunciado ou aumento da complexidade articulatória de sílaba / palavra	Aumento das distorções de som ou substituições de som distorcidas com aumento do tamanho do enunciado ou aumento da complexidade articulatória de sílaba / palavra	Increased sound distortions or distorted sound substitutions with increased utterance length or increased syllable/word articulatory complexity	Aumento das distorções de som ou substituições de som distorcidas com aumento do tamanho do enunciado ou aumento da complexidade articulatória de sílaba / palavra
5- Syllable segmentation within words > 1 syllable (Brief silent interval between syllables and/or inappropriate equalized stress across syllables)	Segmentação de sílaba dentro de palavras > 1 sílaba (breve intervalo silencioso entre as sílabas e / ou tonicidade inadequada entre as sílabas)	Segmentação de sílaba dentro de palavras > 1 sílaba (breve intervalo silencioso entre as sílabas e / ou tonicidade inadequada entre as sílabas)	Segmentação de sílaba dentro de palavras > 1 sílaba (breve intervalo silencioso entre as sílabas e / ou tonicidade inadequada entre as sílabas)	Syllable segmentation within words > 1 syllable (Brief silent interval between syllables and/or inappropriate equalized stress across syllable)	Segmentação de sílaba dentro de palavras > 1 sílaba (breve intervalo silencioso entre as sílabas e / ou tonicidade inadequada entre as sílabas)
6- Syllable segmentation across words in phrases/sentences (Increased inter-word intervals and/or inappropriate equalized stress across words)	Segmentação de sílaba entre palavras em frases / sentenças (aumento dos intervalos entre palavras e / ou tonicidade inadequada entre palavras)	Segmentação de sílaba entre palavras em frases / sentenças (aumento dos intervalos entre palavras e / ou tonicidade inadequada entre palavras)	Segmentação de sílaba entre palavras em frases / sentenças (aumento dos intervalos entre palavras e / ou tonicidade inadequada entre palavras)	Syllable segmentation across words in phrases/sentences (Increased inter-word intervals and/or inappropriate equalized stress across words)	Segmentação de sílaba entre palavras em frases / sentenças (aumento dos intervalos entre palavras e / ou tonicidade inadequada entre palavras)
7- Slow overall speech rate (apart from pauses for word retrieval and/or verbal formulation)	Velocidade geral de fala lenta (exceto pausas para recuperação de palavras e / ou formulação verbal	Velocidade geral de fala lenta (exceto pausas para recuperação de palavras e / ou formulação verbal)	Velocidade geral de fala lenta (exceto pausas para recuperação de palavras e / ou formulação verbal	Slow overall speech rate (apart from pauses for word retrieval and/or verbal formulation)	Velocidade geral de fala lenta (exceto pausas para recuperação de palavras e / ou formulação verbal
8- Lengthened vowel &/or consonant segments independent of overall slow speaking rate	Vogais e / ou segmentos consonantais alongados, independentemente da velocidade geral de fala lenta	Vogais e / ou segmentos consonantais alongados, independentemente da velocidade geral de fala lenta	Vogais e / ou segmentos consonantais alongados, independentemente da velocidade geral de fala lenta quando fala	Lengthened vowel &/or consonant segments independent of overall slow speaking rate	Vogais e / ou segmentos consonantais alongados, independentemente da velocidade geral de fala lenta
9- RATE ONLY FOR AMRs (alternating motion rates, as in rapid repetition of “puh puh puh”): Slow and/or off-target (in place, manner, and/or voicing) 0= AMRs normal; 1= rare and mild, 2= frequent but mild; 3 = moderate, 4 = severe	AVALIAÇÃO SOMENTE PARA AMRs (avaliações de movimento alternado, como na repetição rápida de “pa pa pa”): Lento e / ou fora do alvo (no ponto, modo e/ou vozeamento) 0 = AMRs normais; 1 = raro e leve, 2 = frequente, mas leve; 3 = moderado, 4 = intenso	AVALIAÇÃO SOMENTE PARA TMFA (avaliações da tarefa motora de fala alternada, como na repetição rápida de “pa pa pa”): Lento e / ou fora do alvo (no ponto, modo e/ou vozeamento) 0 = TMFA normais; 1 = raro e leve, 2 = frequente, mas leve; 3 = moderado, 4 = intenso	AVALIAÇÃO SOMENTE PARA TMFA (avaliações da tarefa motora de fala alternada, como na repetição rápida de “pa pa pa”): Lento e / ou fora do alvo (no ponto, modo e/ou vozeamento) 0 = TMFA normais; 1 = raro e leve, 2 = frequente, mas leve; 3 = moderado, 4 = intenso	RATE ONLY FOR AMRs (alternating motion rates, as in rapid repetition of “puh puh puh”): Slow and/or off-target (in place, manner, and/or voicing) 0= AMRs normal; 1= rare and mild, 2= frequent but mild; 3 = moderate, 4 = severe	AVALIAÇÃO SOMENTE PARA TMFA (avaliações da tarefa motora de fala alternada, como na repetição rápida de “pa pa pa”): Lento e / ou fora do alvo (no ponto, modo e/ou vozeamento) 0 = TMFA normais; 1 = raro e leve, 2 = frequente, mas leve; 3 = moderado, 4 = intenso
10- RATE ONLY FOR SMRs (sequential motion rates, as in rapid repetition of “puh tuhkuh”): Slow (gaps within sequences), segmented (gaps between sequences), incorrectly sequenced, and/or off-target (in place, manner, and/or voicing) 0= SMRs normal; 1= any one of the listed features, 2= any two of the listed features; 3 = any three of the listed features, 4 = four of the listed features	AVALIAÇÃO SOMENTE PARA SMRs (avaliações de movimento sequencial, como na repetição rápida de “pa ta ka”): Lento (lacunas dentro das sequências), segmentado (lacunas entre as sequências), sequenciado incorretamente e / ou fora do alvo (no ponto, modo e/ou vozeamento) 0 = SMRs normal; 1 = qualquer um dos recursos listados, 2 = quaisquer dois dos recursos listados, 3 = quaisquer três dos recursos listados, 4 = quatro dos recursos listados	AVALIAÇÃO SOMENTE PARA TMFS (avaliações da tarefa motora de fala sequenciada, como na repetição rápida de “pa ta ka”): Lento (intervalos dentro das sequências), segmentado (intervalos entre as sequências), sequenciado incorretamente e / ou fora do alvo (no ponto, modo e/ou vozeamento) 0 =TMFS normal; 1 = qualquer um dos recursos listados, 2 = quaisquer dois dos recursos listados, 3 = quaisquer três dos recursos listados, 4 = quatro dos recursos listados	AVALIAÇÃO SOMENTE PARA TMFS (avaliações da tarefa motora de fala sequenciada, como na repetição rápida de “pa ta ka”): Lento (intervalos dentro das sequências), segmentado (intervalos entre as sequências), sequenciado incorretamente e / ou fora do alvo (no ponto, modo e/ou vozeamento) 0 =TMFS normal; 1 = qualquer um dos recursos listados, 2 = quaisquer dois dos recursos listados, 3 = quaisquer três dos recursos listados, 4 = quatro dos recursos listados	RATE ONLY FOR SMRs (sequential motion rates, as in rapid repetition of “puh tuhkuh”): Slow (gaps within sequences), segmented (gaps between sequences), incorrectly sequenced, and/or off-target (in place, manner, and/or voicing) 0= SMRs normal; 1= any one of the listed features, 2= any two of the listed features; 3 = any three of the listed features, 4 = four of the listed features	AVALIAÇÃO SOMENTE PARA TMFS (avaliações da tarefa motora de fala sequenciada, como na repetição rápida de “pa ta ka”): Lento (intervalos dentro das sequências), segmentado (intervalos entre as sequências), sequenciado incorretamente e / ou fora do alvo (no ponto, modo e/ou vozeamento) 0 =TMFS normal; 1 = qualquer um dos recursos listados, 2 = quaisquer dois dos recursos listados, 3 = quaisquer três dos recursos listados, 4 = quatro dos recursos listados
11- One or both of the following: Consistently reduced words per breath group during phrase/sentence production relative to maximum vowel duration; reduced # of AMR repetitions per breath group in the absence of decreased respiratory capacity. Score on average number of syllables/repetitions per breath group across tasks: 0 = more than 7; 1= 6-7; 2 = 4-5; 3 = 3-4; 4 = 2 orless	Um ou ambos dos seguintes: Palavras consistentemente reduzidas por grupo de respiração durante a produção de frase / frase em relação à duração máxima da vogal; número reduzido de repetições de AMR por grupo de respiração na ausência de capacidade respiratória diminuída. Pontuação no número médio de sílabas / repetições por grupo de respiração nas tarefas: 0 = mais de 7; 1 = 6-7; 2 = 4-5; 3 = 3-4; 4 = 2 ou menos	Um ou ambos dos seguintes: Palavras consistentemente reduzidas por grupo respiratório durante a produção de frase / frase em relação à duração máxima da vogal; número reduzido de repetições de TMFS por grupo respiratório, na ausência de capacidade respiratória diminuída. Pontuação no número médio de sílabas / repetições por grupo de respiração nas tarefas: 0 = mais de 7; 1 = 6-7; 2 = 4-5; 3 = 3-4; 4 = 2 ou menos	Um ou ambos dos seguintes: Palavras consistentemente reduzidas por grupo respiratório durante a produção de frase / frase em relação à duração máxima da vogal; número reduzido de repetições de TMFS por grupo respiratório, na ausência de capacidade respiratória diminuída. Pontuação no número médio de sílabas / repetições por grupo de respiração nas tarefas: 0 = mais de 7; 1 = 6-7; 2 = 4-5; 3 = 3-4; 4 = 2 ou menos	One or both of the following: Consistently reduced words per breath group during phrase/sentence production relative to maximum vowel duration; reduced # of AMR repetitions per breath group in the absence of decreased respiratory capacity. Score on average number of syllables/repetitions per breath group across tasks: 0 = more than 7; 1= 6-7; 2 = 4-5; 3 = 3-4; 4 = 2 orless	Um ou ambos dos seguintes: Palavras consistentemente reduzidas por grupo respiratório durante a produção de frase / frase em relação à duração máxima da vogal; número reduzido de repetições de TMFS por grupo respiratório, na ausência de capacidade respiratória diminuída. Pontuação no número médio de sílabas / repetições por grupo de respiração nas tarefas: 0 = mais de 7; 1 = 6-7; 2 = 4-5; 3 = 3-4; 4 = 2 ou menos
12- Silent articulatory groping	Silêncio articulatório nas tentativas	Silêncio articulatório nas tentativas	Silêncio articulatório nas tentativas	Silent articulatory groping	Silêncio articulatório nas tentativas
13- Audible false starts/restarts or groping including sound repetitions, excluding fillers and unambiguous semantic false starts (e.g., spoo…fork)	Inícios / reinícios falsos audíveis ou nas tentativas incluindo repetições de som, excluindo enchimentos e falsos inícios semânticos inequívocos (ex.: colheee... garfo)	Inícios / reinícios falsos audíveis ou nas tentativas incluindo repetições de som, excluindo pausas preenchidas e falsos inícios semânticos inequívocos (ex.: colheee... garfo)	Inícios / reinícios falsos audíveis ou nas tentativas incluindo repetições de som, excluindo pausas preenchidas e falsos inícios semânticos inequívocos (ex.: colheee... garfo)	Audible false starts/restarts or groping including sound repetitions, excluding fillers and unambiguous semantic false starts (e.g., spoo…fork)	Inícios / reinícios falsos audíveis ou nas tentativas incluindo repetições de som, excluindo pausas preenchidas e falsos inícios semânticos inequívocos (ex.: colheee... garfo)

Os coeficientes de correlação intraclasse (Tabela 1) variaram entre 0,83 e 0,94. Os itens 1, 5, 6,9, 10 e 12 obtiveram valores superiores a 0,9. Os demais itens apresentaram valores entre 0,8 e 0,9. Dessa forma, o valor elevado do CCI para a análise dos itens sugere que a variabilidade entre as respostas dos avaliadores foi baixa, trazendo resultado positivo para a análise de concordância.

**Tabela 1:** Análise do Coeficiente de Correlação Intraclasse - CCI dos itens da *Apraxia of Speech Rating Scale 3.5* traduzida e adaptada para o português brasileiro

**ITENS**	**CCI**	**Intervalo de confiança - 95%**	
		**Limite Inferior**	**Limite Superior**
Distorções de som (excluindo substituições distorcidas ou adições distorcidas)	0,91	0,71	0,99
Substituições de som distorcido	0,88	0,64	0,99
Adições de som distorcido (incluindo vogal intrusiva)	0,83	0,53	0,99
Aumento das distorções de som ou substituições de som distorcidas com aumento do tamanho do enunciado ou aumento da complexidade articulatória de sílaba / palavra	0,87	0,62	0,99
Segmentação de sílaba dentro de palavras > 1 sílaba (breve intervalo silencioso entre as sílabas e / ou tonicidade inadequada entre as sílabas)	0,94	0,80	0,99
Segmentação de sílaba entre palavras em frases / sentenças (aumento dos intervalos entre palavras e / ou tonicidade inadequada entre palavras	0,90	0,69	0,99
Velocidade geral de fala lenta (exceto pausas para recuperação de palavras e / ou formulação verbal)	0,84	0,57	0,99
Vogais e / ou segmentos consonantais alongados, independentemente da velocidade geral de fala lenta	0,86	0,59	0,99
AVALIAÇÃO SOMENTE PARA TMFA (avaliações da tarefa motora de fala alternada, como na repetição rápida de “pa pa pa”): Lento e / ou fora do alvo (no ponto, modo e/ou vozeamento) 0 = TMFA normais; 1 = raro e leve, 2 = frequente, mas leve; 3 = moderado, 4 = intenso	0,92	0,74	0,99
AVALIAÇÃO SOMENTE PARA TMFS (avaliações da tarefa motora de fala sequenciada, como na repetição rápida de “pa ta ka”): Lento (intervalos dentro das sequências), segmentado (intervalos entre as sequências), sequenciado incorretamente e / ou fora do alvo (no ponto, modo e/ou vozeamento) 0 =TMFS normal; 1 = qualquer um dos recursos listados, 2 = quaisquer dois dos recursos listados, 3 = quaisquer três dos recursos listados, 4 = quatro dos recursos listados	0,93	0,75	0,99
Um ou ambos dos seguintes: Palavras consistentemente reduzidas por grupo respiratório durante a produção de frase / frase em relação à duração máxima da vogal; número reduzido de repetições de TMFS por grupo respiratório, na ausência de capacidade respiratória diminuída. Pontuação no número médio de sílabas / repetições por grupo de respiração nas tarefas: 0 = mais de 7; 1 = 6-7; 2 = 4-5; 3 = 3-4; 4 = 2 ou menos	0,87	0,62	0,99
Silêncio articulatório nas tentativas	0,91	0,70	0,99
Inícios / reinícios falsos audíveis ou nas tentativas incluindo repetições de som, excluindo pausas preenchidas e falsos inícios semânticos inequívocos (ex.: colheee... garfo)	0,88	0,64	0,99
**Total**	**0,85**	**0,78**	**0,90**

Na Tabela 2 são apresentados os resultados quanto à análise pelo IVC-I e IVC-T para análise da relevância e da viabilidade dos itens.

**Tabela 2:** Análise do Índice de Validade de Conteúdo - IVC dos itens do *Apraxia of Speech Rating Scale 3.5* traduzida e adaptada para o português brasileiro

**ITENS**	**IVC -I**	
	**Relevância**	**Viabilidade**
Distorções de som (excluindo substituições distorcidas ou adições distorcidas)	1,000	0,944
Substituições de som distorcido	1,000	0,889
Adições de som distorcido (incluindo vogal intrusiva)	0,889	0,833
Aumento das distorções de som ou substituições de som distorcidas com aumento do tamanho do enunciado ou aumento da complexidade articulatória de sílaba / palavra	0,944	0,944
Segmentação de sílaba dentro de palavras > 1 sílaba (breve intervalo silencioso entre as sílabas e / ou tonicidade inadequada entre as sílabas)	1,000	1,000
Segmentação de sílaba entre palavras em frases / sentenças (aumento dos intervalos entre palavras e / ou tonicidade inadequada entre palavras)	0,944	0,944
Velocidade geral de fala lenta (exceto pausas para recuperação de palavras e / ou formulação verbal)	0,944	0,944
Vogais e / ou segmentos consonantais alongados, independentemente da velocidade geral de fala lenta	0,944	0,889
AVALIAÇÃO SOMENTE PARA TMFA (avaliações da tarefa motora de fala alternada, como na repetição rápida de “pa pa pa”): Lento e / ou fora do alvo (no ponto, modo e/ou vozeamento) 0 = TMFA normais; 1 = raro e leve, 2 = frequente, mas leve; 3 = moderado, 4 = intenso	1,000	1,000
AVALIAÇÃO SOMENTE PARA TMFS (avaliações da tarefa motora de fala sequenciada, como na repetição rápida de “pa ta ka”): Lento (intervalos dentro das sequências), segmentado (intervalos entre as sequências), sequenciado incorretamente e / ou fora do alvo (no ponto, modo e/ou vozeamento) 0 =TMFS normal; 1 = qualquer um dos recursos listados, 2 = quaisquer dois dos recursos listados, 3 = quaisquer três dos recursos listados, 4 = quatro dos recursos listados	1,000	1,000
Um ou ambos dos seguintes: Palavras consistentemente reduzidas por grupo respiratório durante a produção de frase / frase em relação à duração máxima da vogal; número reduzido de repetições de TMFS por grupo respiratório, na ausência de capacidade respiratória diminuída. Pontuação no número médio de sílabas / repetições por grupo de respiração nas tarefas: 0 = mais de 7; 1 = 6-7; 2 = 4-5; 3 = 3-4; 4 = 2 ou menos	0,944	0,944
Silêncio articulatório nas tentativas	1,000	1,000
Inícios / reinícios falsos audíveis ou nas tentativas incluindo repetições de som, excluindo pausas preenchidas e falsos inícios semânticos inequívocos (ex.: colheee... garfo)	1,000	0,944
**Total**	**0,97**	**0,94**

Os coeficientes calculados para relevância obtiveram valores iguais ou superiores a 0,89, já para viabilidade, obtiveram valores iguais ou superiores a 0,83 (Item 3). Desta forma os valores calculados para o IVC sugerem excelentes parâmetros para as análises de relevância e de viabilidade.

## DISCUSSÃO

A metodologia utilizada neste estudo viabilizou a tradução e adaptação transcultural da escala de avaliação *Apraxia of Speech Scale* 3.5 (*ASRS*) para o português brasileiro. O *ASRS* 3.5^(17)^ é uma escala de avaliação clínica da apraxia de fala adquirida com adequadas propriedades psicométricas e com a conclusão do processo de validação será capaz de contribuir na identificação da presença da apraxia de fala, como na classificação da frequência\severidade das manifestações na fala e para ter um diagnóstico diferencial entre afasia, apraxia de fala adquirida e disartria.

Para validação original da *ASRS* 3.5^(17)^, utilizou-se como padrão de referência a avaliação de três clínicos experientes, que tiveram acesso às tarefas de fala de 133 pacientes previamente diagnosticados com apraxia de fala e/ou afasia, incluindo: resposta a perguntas, repetição de palavras e frases, nomeação, tarefas de preenchimento de sentenças, fala espontânea, tarefa motora de fala alternada e tarefa motora de fala sequenciada. A partir do julgamento perceptual dessas tarefas os avaliadores estabeleciam o diagnóstico de afasia (subespecificando a sua tipologia) ou apraxia de fala e a severidade das manifestações encontradas^(17)^.

Na sequência, esse diagnóstico recebia uma confirmação por consenso por dois outros clínicos não envolvidos com o processo e que também tiveram acesso às amostras de fala. Os resultados da *ASRS* 3.5 foram, então, comparados em relação ao resultado da avaliação citada. Pacientes com apraxia de fala receberam escores mais elevados em relação aos não apráxicos, estabelecendo-se o valor de corte de oito pontos, que obteve sensibilidade de 96% e especificidade de 100% na identificação da apraxia. Houve uma correlação positiva forte (r=0,88) entre o escore da *ASRS* 3.5 e a severidade indicada previamente pelos clínicos no padrão de referência, o que reforça o potencial da escala em estabelecer a severidade do quadro apráxico^(17)^.

Desta forma, observou-se o potencial para utilização da *ASRS* 3.5 no ambiente clínico, para identificar e quantificar a apraxia de fala de maneira geral. Espera-se, então, que seja possível observar esse potencial no português brasileiro, com a continuidade do estudo de validação iniciado neste estudo. Com a utilização da escala *ASRS* 3.5 haverá benefícios para profissionais que trabalham com pacientes com alterações neurológicas adquiridas, favorecendo a identificação precoce do transtorno de fala, a tipologia e severidade das características, e a seleção de conduta terapêutica fonoaudiológica de forma mais assertiva.

Dessa forma, verificou-se que o processo de tradução, adaptação transcultural e retrotradução da *ASRS* 3.5 para o português brasileiro manteve a veracidade das informações da versão original. Sugere-se que, com este processo, as discrepâncias linguísticas e culturais sejam solucionadas, pois é imprescindível que o contexto populacional seja considerado neste momento de tradução^(22-23)^.

Durante a tradução e sínteses, houve a busca pela seleção de palavras e frases adequadas ao contexto cultural do português brasileiro e pela manutenção do sentido original da escala. Dessa forma, destaca-se a importância do comitê de especialistas para garantir as equivalências cultural e linguística do *ASRS* 3.5. Nesse sentido, os valores adequados de IVC-I e IVC-Q reforçaram a relevância e viabilidade da escala na cultura brasileira.

No que se refere ao item acerca de “distorções de som”, apresentou relevância, viabilidade e adequação com parâmetros excelentes, seu valor do CCI foi considerado superior (0,91). A distorção do som é uma alteração fonética que não envolve regras fonológicas da língua. Ela é caracterizada por dificuldades motoras envolvidas na produção dos sons, como: imprecisão de local, tempo, pressão e velocidade resultando um som não padrão de fala^(24)^.

E em relação ao item de “substituições de som distorcido”, foi julgado relevante, viável e adequado. As substituições de som distorcido são manifestações frequentes em quadros de apraxia de fala. Uma vez que o indivíduo apráxico substitui os sons que ele distorceu previamente, apresentando, dessa forma, erros inconsistentes no fluxo da fala^(2,25)^.

O item “aumento das distorções de som ou substituições de som distorcido com aumento do tamanho do enunciado ou aumento da complexidade articulatória de sílaba/palavra” foi julgado relevante, viável e adequado. Os indivíduos apráxicos podem apresentar mais dificuldades com o aumento da complexidade articulatória^(25)^.

No que se refere a “segmentação de sílaba dentro de palavras”, é uma característica que reflete uma dificuldade na coarticulação das sílabas e segmentação frequente, o que pode resultar em falhas prosódicas. Esse item pode ser quantificado, em que a taxa e a duração das produções de palavras e sentenças podem ser valiosas no diagnóstico, com alto valor preditivo da apraxia de fala^(26)^.

Os itens “segmentação de sílaba entre palavras em frases/sentenças (aumento dos intervalos entre palavras e /ou tonicidade inadequada entre palavra”, “velocidade geral de fala lenta” e “vogais e/ou segmentos consonantais alongados, independentemente da velocidade geral de fala lenta” foram julgados relevantes, viáveis e adequados. A apraxia afeta a produção dos sons de fala e a organização destes na formação de sílabas e de palavras^(1)^. Dessa forma, o indivíduo apráxico pode apresentar fala mais lenta e segmentada, pois falhas no planejamento motor dificultam o encadeamento das sílabas e palavras e falhas nos padrões de entonação, ritmo e melodia da fala^(27)^.

O item relacionado a “avaliação somente para as avaliações da tarefa motora de fala alternada e sequenciada” foi julgado como viável, relevante e adequada. Essas duas tarefas são importantes para o diagnóstico de apraxia, pois são utilizadas para emitir segmentos de fala em relação aos parâmetros de velocidade, intensidade, ritmo, precisão, duração da emissão e coeficientes de variação, parâmetros esses que são avaliados e geralmente alterados na apraxia^(3,26)^. Devido a dificuldades na coordenação pneumofonoarticulatória e na programação motora de programas musculares relacionados à respiração e à fala^(28)^.

Portanto, o item relacionado a “existência de palavras consistentemente reduzidas por grupo respiratório durante a produção de frase/frase em relação à duração máxima da vogal; número reduzido de repetições de tarefa motora de fala sequenciada por grupo respiratório, na ausência de capacidade respiratória diminuída” também foi julgado relevante e viável.

O item “silêncio articulatório nas tentativas” foi julgado relevante, viável e adequado. Pacientes apráxicos, apresentam esforços para achar a postura certa da articulação; geralmente as mímicas faciais, são cercadas por movimentos silenciosos dos lábios de forma contorcida e forçada, apresentando um tateio articulatório e esforço produtivo^(29)^.

O item “inícios / reinícios falsos audíveis ou nas tentativas incluindo repetições de som, excluindo pausas preenchidas e falsos inícios semânticos” foi julgado relevante, viável e adequado. Essa característica ocorre devido a uma interrupção da programação motora, incorporando erros em comando motor que resulta em reinícios e repetições iniciais de sílabas^(30)^.

Em suma, os valores calculados pelo o CCI e IVC tiveram excelentes parâmetros no quesito de adequado, relevante e viável. O processo de validação da escala *ASRS* 3.5 terá continuidade a partir da versão traduzida e adaptada**.**

## CONCLUSÃO

A versão brasileira da *Apraxia of Speech Rating Scale 3.5* apresenta equivalência semântica, idiomática, experiencial, conceitual e sintática/gramatical em relação ao original. Dessa forma, está apta para as próximas etapas de validação.

## Apêndice A Escala de avaliação da apraxia de fala. Versão traduzida e adaptada transculturalmente do *Apraxia of Speech Rating Scale 3.5* para o português brasileiro

**Escala de Avaliação da Apraxia de Fala**

**-*Apraxia of Speech Rating Scale 3.5* -**

**Nome completo: ______________________________________________________ _________ Idade: _____________**

**Data de nascimento: ___/___/___ Data da avaliação: ___/___/___ Examinador: ___________________________________**

**Table:**

**ESCORE**	**0**	**1**	**2**	**3**	**4**
**DESCRIÇÃO**	**Não observado em nenhuma tarefa**	**Raro**	**Frequente, mas não pervasivo**	**Ocorre quase sempre, em grau menos severo**	**Ocorre quase sempre, em grau mais severo**
**DIRETRIZES**	Menos de uma ocorrência	Mais de uma ocorrência, mas em menos de 20% das palavras	Observado em cerca de 20-50% das palavras	Observado na maioria das palavras	Observado em quase todas as palavras
**EXCEÇÕES**				Pontuação não superior a “2” se presente apenas durante a repetição	Pontuação “4” se a inteligibilidade for mais do que ligeiramente reduzida
Desempenho na TMFA e na TMFS considerados para os itens 9-11					
**CARACTERÍSTICAS FONÉTICAS**					
**1** ^APRAXIA/DISARTRIA^	Distorções de som (excluindo substituições distorcidas ou adições distorcidas)				
**2** ^APRAXIA^	Substituições de som distorcido				
**3** ^APRAXIA^	Adições de som distorcido (incluindo vogal intrusiva)				
**4** ^APRAXIA^	Aumento das distorções de som ou substituições de sons distorcidos com aumento do tamanho do enunciado ou aumento da complexidade articulatória de sílaba / palavra				
CARACTERÍSTICAS PROSÓDICAS					
**5** ^APRAXIA/DISARTRIA^	Segmentação de sílaba dentro de palavras > 1 sílaba (breve intervalo silencioso entre as sílabas e / ou tonicidade inadequada entre as sílabas)				
**6** ^APRAXIA/DISARTRIA^	Segmentação de sílaba entre palavras em frases / sentenças (aumento dos intervalos entre palavras e / ou tonicidade inadequada entre palavras)				
**7** ^APRAXIA/DISARTRIA^	Velocidade geral de fala lenta (exceto pausas para recuperação de palavras e / ou formulação verbal)				
**8** ^APRAXIA/DISARTRIA^	Vogais e / ou segmentos consonantais alongados, independentemente da velocidade geral de fala lenta				
OUTROS					
**9** ^APRAXIA/DISARTRIA/AFASIA^	AVALIAÇÃO SOMENTE PARA TMFA (avaliações da tarefa motora de fala alternada, como na repetição rápida de “pa pa pa”): Lento e / ou fora do alvo (no ponto, modo e/ou vozeamento) 0 = TMFA normais; 1 = raro e leve, 2 = frequente, mas leve; 3 = moderado, 4 = intenso				
**10** ^APRAXIA/DISARTRIA/AFASIA^	AVALIAÇÃO SOMENTE PARA TMFS (avaliações da tarefa motora de fala sequenciada, como na repetição rápida de “pa ta ka”): Lento (intervalos dentro das sequências), segmentado (intervalos entre as sequências), sequenciado incorretamente e / ou fora do alvo (no ponto, modo e/ou vozeamento) 0 =TMFS normal; 1 = qualquer um dos recursos listados, 2 = quaisquer dois dos recursos listados, 3 = quaisquer três dos recursos listados, 4 = quatro dos recursos listados				
**11** ^APRAXIA^	Um ou ambos dos seguintes: Palavras consistentemente reduzidas por grupo respiratório durante a produção de frase / frase em relação à duração máxima da vogal; número reduzido de repetições de TMFS por grupo respiratório, na ausência de capacidade respiratória diminuída. Pontuação no número médio de sílabas / repetições por grupo de respiração nas tarefas: 0 = mais de 7; 1 = 6-7; 2 = 4-5; 3 = 3-4; 4 = 2 ou menos				
**12** ^APRAXIA/AFASIA^	Silêncio articulatório nas tentativas				
**13** ^APRAXIA/DISARTRIA/AFASIA^	Inícios / reinícios falsos audíveis ou nas tentativas incluindo repetições de som, excluindo pausas preenchidas e falsos inícios semânticos inequívocos (ex.: colheee... garfo)				
**ESCORE TOTAL**					

**Legenda:** TMFA = Tarefa motora de fala alternada; TMFS = Tarefa motora de fala sequenciada; APRAXIA = Características distintivas primárias (rara sobreposição com disartria ou afasia); APRAXIA/DISARTRIA = Características distintivas, a menos que haja disartria; APRAXIA/AFASIA = Características distintivas, a menos que haja afasia; APRAXIA/DISARTRIA/AFASIA = Característica distintiva, a menos que afasia e/ou disartria estejam presentes

Tarefas de fala indicadas para a avaliação: Fala espontânea, Conversação, Descrição de imagem/Discurso Narrativo Oral, Repetição de palavras e frases, Tarefa motora de fala alternada e Tarefa motora de fala sequenciada
